# Microscopic and neuroendoscopic treatment of a large ruptured supratentorial dermoid cyst with extensive dissemination: a case report and literature review

**DOI:** 10.3389/fonc.2024.1468622

**Published:** 2024-10-14

**Authors:** Yuhang Zhang, Tingzhen Deng, Zhi Wu, Haijun Yang, Xingyuan Ma, Yatao Wang, Ruiwen Ding, Haotian Li, Dawen Wang, Maohua Zheng

**Affiliations:** ^1^ The First School of Clinical Medicine, Lanzhou University, Lanzhou, China; ^2^ Department of Neurosurgery, The First Hospital of Lanzhou University, Lanzhou, China

**Keywords:** dermoid cyst, ruptured, microscopic surgery, neuroendoscopy, case report

## Abstract

**Introduction and importance:**

Intracranial dermoid cysts are rare, constituting 0.04% to 0.6% of all intracranial tumors. They often arise from ectodermal cells trapped during neural tube formation. We report a case of spontaneous rupture of a large tentorial epithelioid cyst, which caused massive dissemination of liquid cholesterol into the subarachnoid cisterns and ventricles.

**Presentation of case:**

A 28-year-old male presented with a two-week history of headache and memory decline. CT and MRI revealed a 9x6 cm lesion in the left frontotemporal region with widespread dissemination of lipid droplets. Surgical resection was performed using a microscope combined with a neuroendoscope. Pathology confirmed a dermoid cyst.

**Clinical discussion:**

Ruptured dermoid cysts can cause significant symptoms due to the dissemination of cyst contents. Imaging is crucial for diagnosis and surgical planning. The combined microscopic and neuroendoscopic approach minimized blind spots and allowed thorough tumor exposure, facilitating complete resection with minimal residual complications. Postoperative outcomes were favorable, with imaging confirming substantial tumor removal and restored cerebrospinal fluid circulation.

**Conclusion:**

Prompt diagnosis and comprehensive surgical intervention are essential for managing ruptured intracranial dermoid cysts. Combined microscopic and neuroendoscopic techniques are effective in achieving extensive resection and reducing complications.

## 1 Introduction

Intracranial dermoid cysts are rare benign space-occupying lesions usually located in the midline, posterior fossa, suprasellar, frontonasal, or temporal-basal regions (1). The cyst wall is a stratified squamous epithelium containing dermal components, including sebaceous glands, sweat glands, and hair follicles (1, 2). These cysts typically accumulate exfoliated epithelium, sebaceous secretions, fat, and hair, leading to slow growth. Common symptoms included headache, seizures, focal neurological dysfunction, and so on. The rupture of dermoid cyst contents can lead to serious complications, including meningitis, hydrocephalus, endocrine disease, and even blindness (3, 4). We report a case of spontaneous rupture of a large tentorial epithelioid cyst in a male patient, which caused massive dissemination of liquid cholesterol into the subarachnoid cisterns and ventricles, blocking cerebrospinal fluid circulation.

This case report has been reported in line with the Surgical CAse REport (SCARE) Criteria (5).

## 2 Case report

A 28-year-old male was admitted to our hospital with a two-week history of headaches mainly located in the left frontotemporal region and memory decline. He denied any history of head trauma, fever, or seizures. Neurological examination revealed a slight decrease in short-term memory but no other focal neurological deficits.

**Figure 1:**
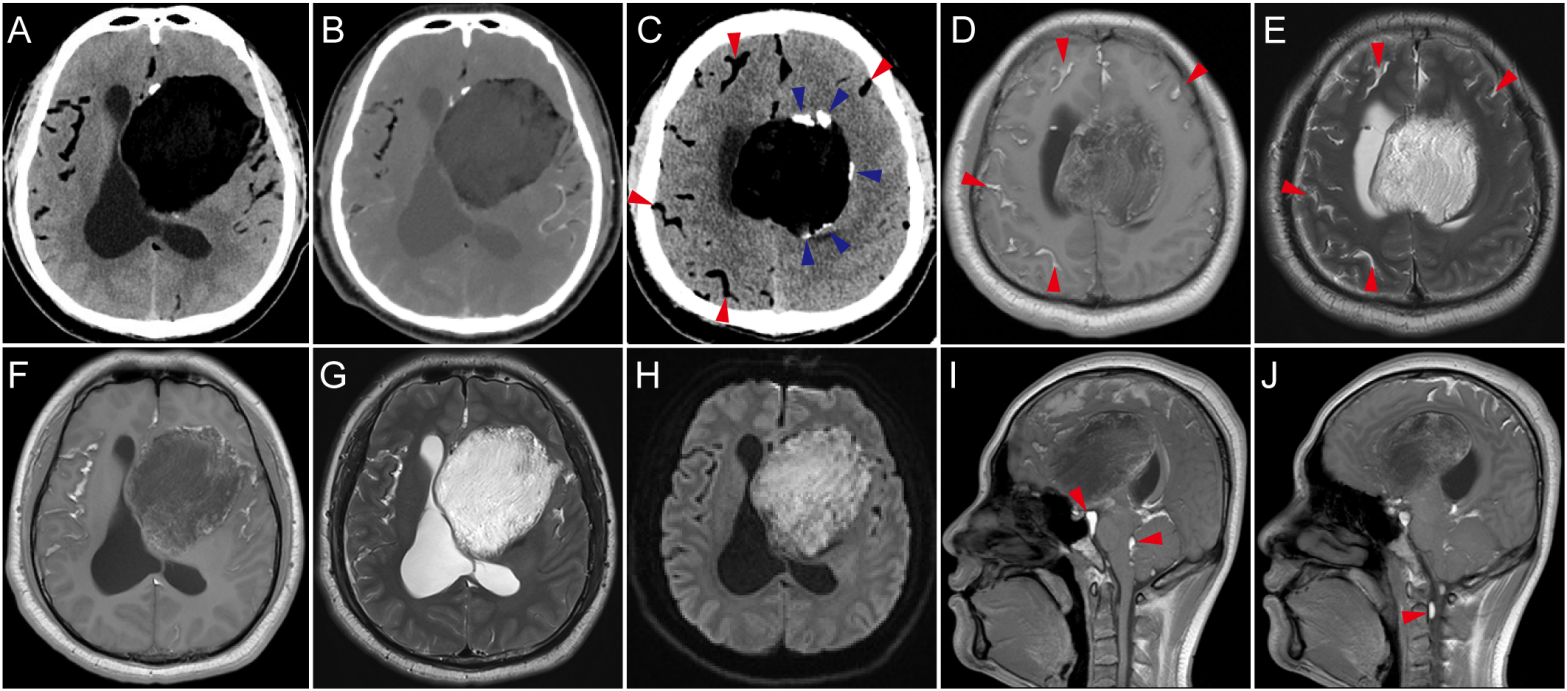
Imaging manifestations. **(A)** Computed tomography (CT) scan showed left frontal temporal there is a huge irregular low-density mass. **(B)** Contrast-enhanced CT showed no enhancement of the mass. **(C)** Calcification of the cyst wall (blue arrow) and hypodense droplets in the subarachnoid spaces (red arrow). **(D, E)** In the axial plane corresponding to **(C)**, the disseminated material in the subarachnoid space showed high signal intensity on both T1-weighted and T2-weighted images. **(F–H)** MRI scans showed the lesion had a heterogeneous signal, predominantly low signal on T1-weighted images with intermixed high signal areas, primarily high signal on T2-weighted images, and irregular hyperintense signals on DWI. Dissemination of cyst contents was visible in the Sylvian fissures, exhibiting high signal on both T1-weighted and T2-weighted images. **(I, J)** On sagittal T1-weighted images, the dissemination is also observed in the fourth ventricle, interpeduncular cistern, ambient cistern, quadrigeminal cistern, and even within the spinal canal, obstructing the cerebrospinal fluid circulation.

Cranial computed tomography (CT) scan showed an approximately 9x6 cm hypodense lesion in the left front temporoparietal lobe (**Figure 1A**), without enhancement after contrast injection (**Figure 1B**). In another section, calcification was observed at the margins of the lesion, and multiple hypodensities were noted in the cerebral sulci and bilateral Sylvian fissures (**Figure 1C**). Magnetic resonance imaging (MRI) confirmed the diffusion of lipids after rupture, which were hyperintense on both T1 and T2-weighted images (**Figures 1D, E**). MRI also revealed a large heterogeneous cystic lesion, which was hypointense mixed with hyperintense on T1-weighted images and hyperintense on T2-weighted images (**Figures 1F, G**), and irregular hyperintense signals on diffusion weighted imaging (DWI) (**Figure 1H**). On sagittal images, lipid droplets were also seen in the fourth ventricle, interpeduncular cistern, ambient cistern, quadrigeminal cistern, and even within the spinal canal (**Figures 1I, J**). The entire subarachnoid space shows diffusely scattered hyperintense fat droplets. These imaging findings are consistent with the characteristics of a ruptured dermoid cyst, with extremely extensive dissemination of its contents, which is quite rare.

**Figure 2:**
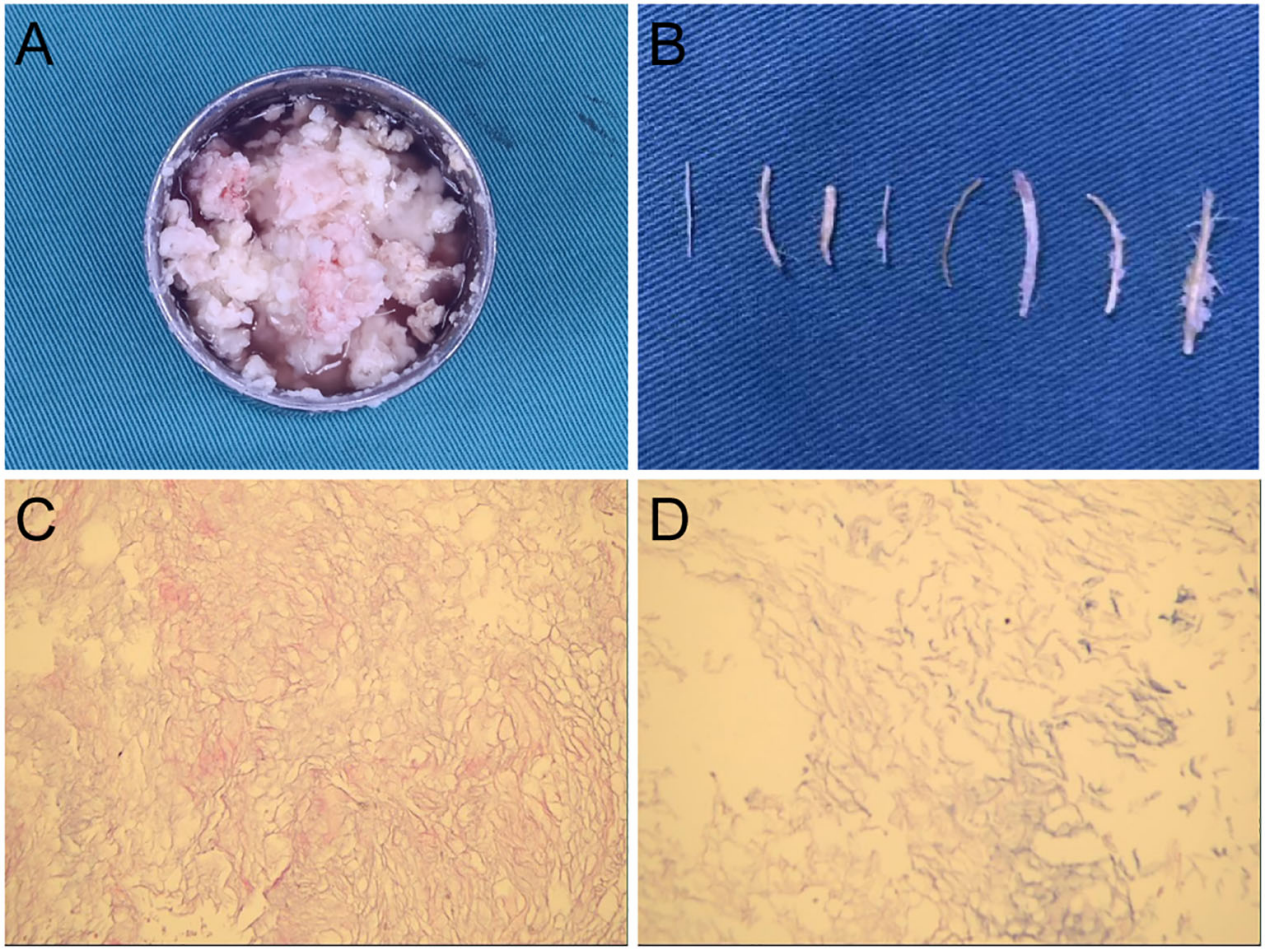
Intraoperative specimen and pathology results. **(A, B)** Yellowish-white material and pilous tissue was found within the cyst. **(C, D)** Histopathological examination confirmed the diagnosis of a dermoid cyst.

**Figure 3:**
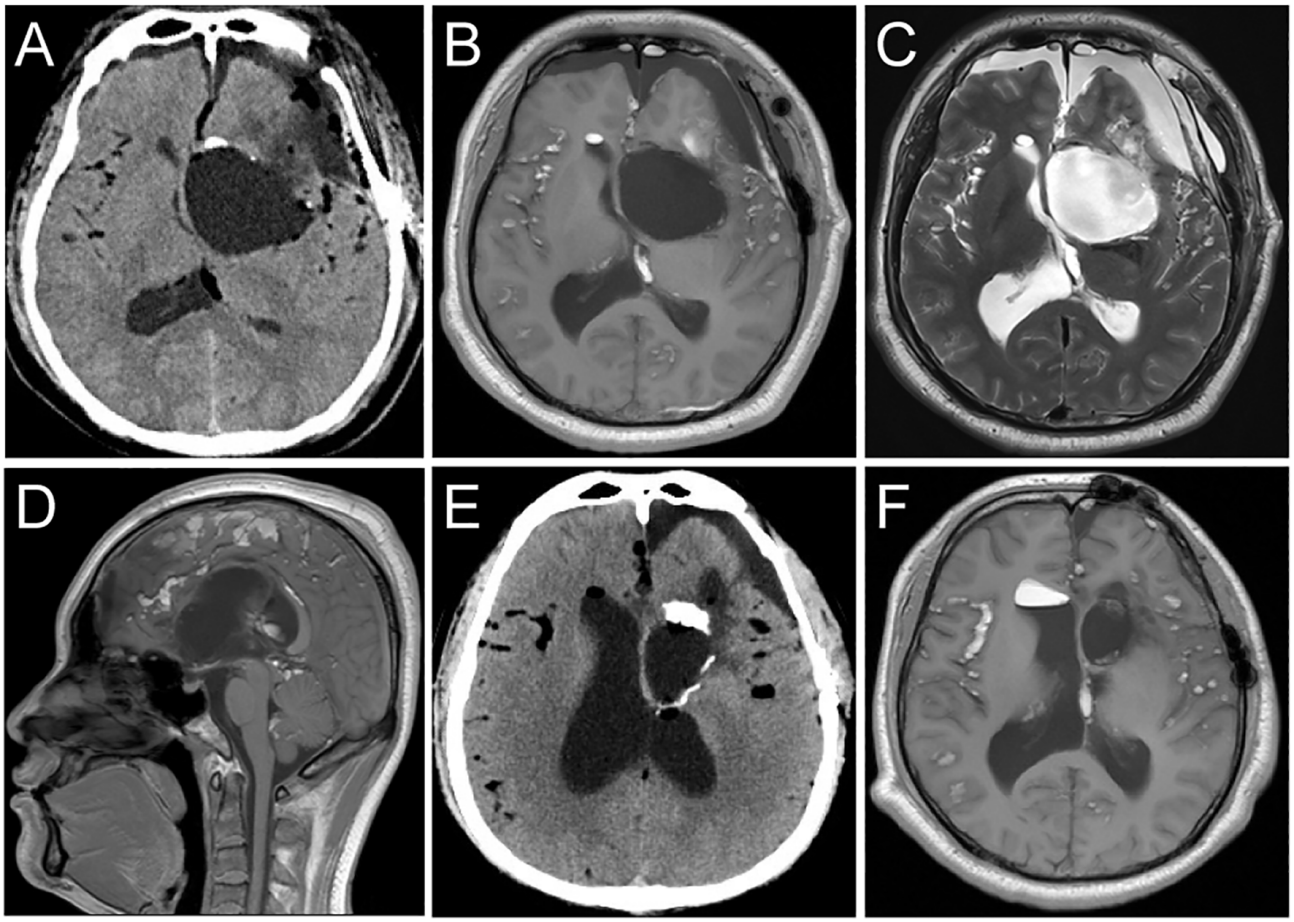
Postoperative follow-up imaging studies. **(A)** CT imaging post-surgery showed that the majority of the dermoid cyst had been completely resected. **(B, C)** T1 and T2 weighted MRI axial images show that the dermoid cyst has been resected. The residual disseminated material in the Sylvian fissure and subarachnoid space has decreased compared to the preoperative images. **(D)** The sagittal T1-weighted image shows restoration of normal cerebrospinal fluid circulation. **(E, F)** CT scans at three months and MRI at one year showed no signs of recurrence, and the majority of the fat droplets had been reduced, some residual fat droplets remain.

The patient underwent a combined microscopic and neuroendoscopic surgical resection of the lesion. After opening the dura mater, small fat droplets were observed in the subarachnoid spaces, similar to the preoperative scan. A large amount of yellowish-white material and pilous tissue was found within the cyst (**Figures 2A, B**). During the surgery, cottonoids were used to cover the surrounding brain tissue, and the subarachnoid space was irrigated to wash out as much cyst dissemination as possible. The cyst wall was carefully dissected from the surrounding brain tissue and removed. Histopathological examination confirmed the diagnosis of a dermoid cyst (**Figures 2C, D**). Postoperative CT showed a significant reduction of the lesion (**Figures 3A, B**), and MRI demonstrated clear cerebrospinal fluid circulation, although some fat droplets persisted in the cerebral sulci (**Figures 3C, D**). Post surgery, the patient’s headache had significantly improved, and he had no other symptoms. At the three-month and one-year follow-ups, the patient reported no discomfort. CT scans at three months and MRI at one year showed no signs of recurrence, and the majority of the fat droplets had been reduced, some residual fat droplets remain (**Figures 3E, F**).

## 3 Discussion

Intracranial dermoid cysts account for 0.04% to 0.6% of all intracranial tumors (1). They typically arise due to incomplete separation of surface ectodermal cells from the neural tube between the third and fifth weeks of gestation, leading to entrapment of these cells within the neural tube. This mechanism explains why dermoid cysts are commonly located in the infratentorial and midline regions (6). However, some lesions are located in supratentorial and in the lateral ventricles due to the displacement of ectodermal cells by the developing neurovascular system, leading to ectopic positioning (7). In this case, the dermoid cyst occurred in the supratentorial front-temporoparietal region, which is relatively rare. The outer capsule of a dermoid cyst consists of dense fibrous connective tissue lined by stratified squamous epithelium. It contains various ectodermal derivatives, including sweat glands, sebaceous glands, hair follicles, squamous epithelium, teeth, and nails (8). The growth of dermoid cysts depends on the accumulation of desquamated keratin and sebaceous gland secretions rather than cell division, thus growing extremely slowly (4). Patients are typically asymptomatic, and symptoms only manifest when the cyst enlarges sufficiently to cause a mass effect, presenting symptoms such as headache, seizures, and focal neurological deficits (9). However, when a dermoid cyst ruptures, either spontaneously or due to external factors, its characteristics differ significantly from those of an unruptured dermoid cyst.

Dermoid cyst rupture is extremely rare and is currently categorized into two main types: spontaneous and non-spontaneous rupture. Spontaneous rupture is the most common type. One proposed explanation for spontaneous rupture involves rapid cyst enlargement due to age-related hormonal changes. However, a retrospective study on dermoid cyst rupture found that ruptures did not predominantly occur among hormonally active adolescents; the average age at rupture was 32 years (10, 11). Non-spontaneous rupture most commonly results from trauma, followed by factors such as cerebral pulsation and head movement (9). In this case, the patient experienced spontaneous rupture without external factors.

After dermoid cyst rupture, keratin and cholesterol breakdown products diffuse into the subarachnoid space and ventricles. The dissemination of cyst contents can cause a spectrum of symptoms such as headache, seizures, hydrocephalus, meningitis, cognitive dysfunction, focal neurological deficits, vasospasm leading to stroke, and even death (1). It is currently unclear whether these clinical manifestations arise immediately after cyst rupture or develop acutely following chronic spread of dermoid contents and inflammation (3). Headache, a primary symptom of dermoid cyst rupture, can manifest in various forms, ranging from intermittent to persistent, with severity from mild to severe (12). Depending on the location and size of the cyst, headaches can occur anywhere and may or may not be associated with meningeal signs. In this case, the patient’s complaint was moderate distending pain on the affected side lasting for two weeks, with no signs of meningeal irritation. We attribute this to the cerebrospinal fluid circulation disorder caused by the widespread dissemination of cyst contents into the subarachnoid space after the rupture or mass effect of the dermoid cyst.

The radiological appearance of dermoid cysts depends on their internal components and whether they have ruptured. On CT, dermoid cysts typically appear as low-density lesions due to their fatty content, occasionally with calcifications visible at the cyst wall and generally without surrounding edema. Unruptured dermoid cysts on MRI containing liquid cholesterol exhibit imaging characteristics similar to fat: high signal on T1-weighted images and variable signal intensity on T2-weighted images due to different contents. DWI often shows high or equal signal intensity (11). A ruptured dermoid cyst may show scattered high signal intensity within the subarachnoid space or ventricles on T1 and T2, primarily attributed to lipid substances, which typically appear as low signal on fat-suppressed sequences (1).

In this case, CT imaging of the dermoid cyst demonstrated typical features, with significant deformation and compression of the ipsilateral ventricle, midline shifts to the right, and shallower sulci and fissures with extensive low-density areas visible inside the cyst, indicating disseminated cyst contents, more prominent in the less compressed sulci of the right hemisphere. MRI revealed high signal intensity in the sulci and fissures on T1 and T2, with liquid fat diffusing outside the cyst, explaining the low signal on T1 within the lesion. These disseminated substances typically can persist for several years without absorption. Previous studies suggested that cyst contents do not further migrate or cause new neurological deterioration after spread. Similar findings were noted during postoperative follow-up in this case. CT at three months and MRI at one year revealed scattered fat droplets without significant repositioning.

However, a recent case report on traumatic dermoid cyst rupture highlighted significant ongoing migration of fat in the subarachnoid space over two years post-rupture (13). Another case report linked repetitive golf swings to dermoid cyst rupture, revealing gravitational migration of fat on MRI due to positional factors (14). Therefore, further follow-up is required to determine whether there will be further displacement of the fat droplets and whether it will result in related symptoms.

Regular imaging surveillance may be a reasonable management for small and asymptomatic lesions, whereas larger lesions causing mass effects typically warrant surgical intervention (15). During surgical treatment of ruptured dermoid cysts, decompression of the lesion is usually necessary to prevent cyst contents from entering the subarachnoid space. Extensive irrigation of the surrounding subarachnoid space is performed to wash out as much cystic material as possible, thereby reducing the incidence of postoperative fat dissemination and aseptic meningitis (1, 2). The residual cyst wall is the most common reason for tumor recurrence; therefore, complete removal of the cyst wall is necessary. If the cyst capsule is tightly adhered to surrounding neurovascular structures, subtotal resection with preservation of adherent tissue should be considered to avoid damaging critical structures and minimize complications. Subtotal resection of the cyst is rarely reported recurrence and progression; most patients have a favorable prognosis (1). Lipid droplets are diffusely distributed in the subarachnoid space after cyst rupture, and total removal of these droplets is difficult. It remains uncertain whether the symptoms caused by effluxion can be relieved by local lesion resection. However, glucocorticoid therapy may be beneficial for symptoms caused by spillage stimulation of surrounding structures (4, 16).

As shown in **Table 1**, we have reviewed research on ruptured epidermoid cysts with documented treatment and follow-up records (1, 17–35). Our findings indicate that most patients experienced symptom relief following surgical resection of the cyst. Notably, there was only one case of recurrence and one case of death, which might be attributed to the surgery being performed in a much earlier time period (17, 25). This underscores that surgical intervention remains the primary treatment for ruptured dermoid cysts. For patients presenting with meningitis as the main symptom, steroid therapy can be an effective alternative if surgery is not performed (36, 37). In cases where patients present with post-rupture hydrocephalus as the predominant symptom, cerebroventricular shunting can provide significant symptomatic relief (21, 28).

**Table 1:** Details of literature review on intracranial dermoid cyst rupture.

Year	First Author	Age (year)/gender	Chief complaint	Location	Treatment	Follow up	Prognosis
1950	Miller	30/M	HA	Right frontal pole	Drainage	few days	died
1977	Maravilla	39/M	HA, Fever, Seizure	Right frontal pole	SR	10 months	well
1981	Ford	26/M	Numbness	Left frontal pole	SR	2 months	well
1982	Mikhael	37/M	Hemiparesis	Planum sphenoidale	SR	2 months	well
1989	Martin	46/F	HA, Nausea	Left frontal horn	VP shunt	11 months	well
1993	Takeuchi	24/F	Olfactory Delusion, Psychotic Symptoms	Suprasellar	SR	24 months	well
2001	Nakamura	15/F	HA	Left temporal lobe	SR	12 months	well
2008	Liu	57/M	Seizure	Right peri-sylvian	SR	61 months	well
2008	Liu	25/F	HA, Visual loss	Anterior cranial base	SR	15 months	well
2008	Liu	36/M	Hydrocephalus, Acute mental	Pineal region	SR	116 months	well
2008	Liu	37/M	HA	Parasellar	SR	2 months	well
2008	Liu	35/M	HA, Diplopia, Facial numbness	Suprasellar	SR	134 months	well
2010	Zheng	32/M	HA, Defects of the visual, Papilledema bitemporal	Suprasellar	SR	6 months	well
2012	Park	28/F	HA	Left frontal lobe	SR	13 months	recurrence
2013	Wang	71/M	HA, Neck stiffness	Sulcus	Steroid	1 week	well
2014	EROL	14/M	Acute onset of psychiatric symptoms	Suprasellar	SR	4 weeks	well
2015	Paik	32/M	HA, Tinnitus	Right cavernous sinus	SR	few days	well
2016	A. Wani	30/M	HA, Vomit, Diplopia	Fourth ventricle	VP shunt	12 months	well
2017	Jin	55/F	Sudden receptive Aphasia	Left temporal lobe ​	Aspirin	1 month	well
2018	Sha	2/M	Eye ptosis, Ophthalmoplegia	Left anterior clinoid	SR, Steroid	3 months	well
2019	Borni	32/M	Seizures, Decrease in visual acuity	Suprasellar	SR	few days	well
2020	Xin	26/F	HA, Seizure	Left temporal fossa	SR, SV	3 months	well
2020	Ochoa	12/M	TN	Meckel’s cave	SR	few days	well
2021	Blitz	30/M	HA, Photophobia, Nausea, Vomiting, vision loss	Suprasellar	Lumbar drainage	6 months	well
2024	Baraya	27/F	HA, Vision change	Suprasellar	SR	2 months	well

M, Male; F, Female; HA, Headache; TN, Trigeminal neuralgia; VP, Ventriculoperitoneal; SR, Surgical resection; SV, Sodium valproate.

Radiotherapy is also a treatment for dermoid cysts, though it is typically reserved for cases of recurrence or malignant transformation. The clinical response of any tumor to radiotherapy depends on the inherent radiosensitivity of the tumor cells during treatment. Radiotherapy may reduce or eliminate the proliferative activity of the cells lining the dermoid cyst, thereby decreasing the rate of cyst content accumulation. Malignant transformation of dermoid cysts into squamous cell carcinoma (SCC) is extremely rare (38), primary intracranial SCC has a poor prognosis, and its management remains controversial. However, studies have shown that when complete tumor resection is possible, followed by radiotherapy, it is the optimal approach for improving patient outcomes (39–41). Radiotherapy, either alone or in combination with subtotal resection, can help reduce recurrence rates associated with incomplete tumor removal, especially in patients where the risks of additional neurosurgical procedures are elevated due to comorbidities (42). There are also reports of radiotherapy being used for intraspinal dermoid cysts (43), as well as for other benign intracranial tumors, such as epidermoid tumors (44), where the combination of surgery and adjuvant therapy has significantly improved survival rates (42).

In this case, due to the large size and deep location of the cyst, we adopted a combined microscopic and neuroendoscopic surgical treatment. Microscopic surgery is renowned for its high precision, offering excellent magnification and clarity, which facilitates the detailed visualization and differentiation of anatomical structures. This high magnification assists surgeons in accurately identifying and isolating pathological tissues. However, the microscope’s field of view is relatively limited, which may result in blind spots when addressing large or irregular lesions, potentially making it difficult to fully observe all aspects of the lesion. Additionally, the fixed perspective of the microscope restricts the ability to view hidden lesions or structures that lie outside its immediate field. In contrast, neuroendoscopy provides a broader field of view and superior illumination, enabling a more comprehensive observation of the lesion and its surrounding neurovascular structures. The close-range visualization offered by neuroendoscopy is advantageous for managing complex cases. Importantly, the use of angled endoscopes allows surgeons to navigate around obstacles and examine areas not visible with a microscope, thereby reducing blind spots. Nevertheless, neuroendoscopy involves a more complex operational technique and generally offers lower magnification compared to microscopy, which can impact the precision of handling fine structures. Combining microscopy and neuroendoscopy optimizes the reduction of blind spots and ensures thorough exposure of tumor regions obstructed by anatomical structures. This integrated approach significantly enhances the likelihood of achieving complete tumor resection. Postoperatively, the patients experienced significant pain relief, and CT imaging showed that the dermoid cyst was mostly resected without complications. One week later, an MRI showed restored patency of the cerebrospinal fluid circulation path and symmetrical ventricular shape, indicating a favorable surgical outcome. While most intracerebral fat deposits in brain sulci persisted, at three-month follow-up, the patient reported minimal discomfort with essentially resolved pain symptoms. CT shows a decrease in lipid deposition compared to before. One year later, the patient reported no specific discomfort. Follow-up MRI showed no recurrence, but lipid deposits were still present.

## 4 Conclusion

Reports of dermoid cysts are uncommon, and ruptured dermoid cysts are rare. This case presents an atypically located, large supratentorial dermoid cyst that ruptured spontaneously, causing extensive lipid dissemination within the subarachnoid space and resulting in symptoms such as headaches and memory impairment. Surgical intervention using a combined microscopic and neuroendoscopic approach proved effective, facilitating thorough resection and minimizing blind spots. Postoperative outcomes were favorable, with significant pain relief and restored cerebrospinal fluid circulation, with follow-up imaging showing no recurrence and decreased lipid deposition. This case underscores the importance of considering surgical resection for large dermoid cysts with mass effect and highlights the utility of combined microscopy and neuroendoscopy for complete cyst removal.

## Data Availability

The original contributions presented in the study are included in the article/supplementary material. Further inquiries can be directed to the corresponding author.
